# Medication errors in emergency departments: is electronic medical record an effective barrier?

**DOI:** 10.31744/einstein_journal/2019GS4282

**Published:** 2019-06-28

**Authors:** Marina Vaidotas, Paula Kiyomi Onaga Yokota, Neila Maria Marques Negrini, Dafne Braga Diamante Leiderman, Valéria Pinheiro de Souza, Oscar Fernando Pavão dos Santos, Nelson Wolosker

**Affiliations:** 1 Hospital Israelita Albert Einstein, São Paulo, SP, Brazil.

**Keywords:** Medication errors, Harm reduction, Medical records systems, computerized, Electronic prescribing, Emergency medical services, Erros de medicação, Redução do dano, Sistemas computadorizados de registros médicos, Prescrição eletrônica, Serviços médicos de emergência

## Abstract

**Objective:**

To compare medication errors in two emergency departments with electronic medical record, to two departments that had conventional handwritten records at the same organization.

**Methods:**

A cross-sectional, retrospective, descriptive, comparative study of medication errors and their classification, according to the National Coordinating Council for Medication Error Reporting and Prevention, associated with the use of electronic and conventional medical records, in emergency departments of the same organization, during one year.

**Results:**

There were 88 events per million opportunities in the departments with electronic medical record and 164 events per million opportunities in the units with conventional medical records. There were more medication errors when using conventional medical record – in 9 of 14 categories of the National Coordinating Council for Medication Error Reporting and Prevention.

**Conclusion:**

The emergency departments using electronic medical records presented lower levels of medication errors, and contributed to a continuous improvement in patients´ safety.

## RESUMO

**Objetivo:**

Comparar os erros de medicações de duas unidades de pronto atendimento que possuíam prontuário eletrônico aos de duas unidades que possuíam prontuário convencional manual em uma mesma instituição.

**Métodos:**

Estudo transversal, retrospectivo, descritivo, que comparou a incidência de erros de medicações e sua classificação, segundo o *National Coordinating Council for Medication Error Reporting and Prevention*, associado ao uso do prontuário eletrônico e do convencional, em unidades de pronto atendimento de uma mesma instituição por um ano.

**Resultados:**

Foram observados 88 eventos por milhão de oportunidades nas unidades com prontuário eletrônico e 164 por milhão de oportunidades nas unidades com prontuário convencional. Houve mais erros de medicações nas unidades com prontuário convencional − em 9 das 14 categorias da *National Coordinating Council for Medication Error Reporting and Prevention* analisadas.

**Conclusão:**

Com a utilização do prontuário eletrônico, as unidades de pronto atendimento apresentaram menores índices de erros de medicações, contribuindo para melhoria continuada na segurança do paciente.

## INTRODUCTION

According to the National Coordinating Council for Medication Error Reporting and Prevention (NCC MERP), medication error (ME) is the mistaken use or even the non-administration of a medication that results in harm to the patient (regardless of severity).^(^ ^1^ ^)^

The events can be linked to professional practice, healthcare products, medical procedures, and prescription systems. There may be errors in prescription items, in communication of the prescription, in the text of the product label, in the package and name of the medication, in its composition, distribution, and administration, in the training of professionals, and in the supervision of medication use.^(^ ^1^ ^)^

According to what is divulged by the Center of Disease Control and Prevention (CDC), in the United States alone, in 2012, events related to ME were responsible for more than 700 thousand cases of emergency and 120 thousand hospital admissions, with an estimated expenditure of more than US$ 3.5 billion, approximately 40.0% of which were avoidable events.^(^ ^2^ ^)^ With the development of new medications and aging of the population, these numbers may grow even more.^(^ ^2^ ^,^ ^3^ ^)^

The use of the Electronic Medical Records (EMR) is a support tool for administration of medications. There is a hypothesis that the EMR acts in preventing failures, with a potential for checking the entire process, from input of an item on prescription to its administration. Any system that increases supervision and checking of each one of these steps has the potential of improving patients´ safety, regardless of complexity of the technological solution adopted in this process.^(^ ^4^ ^–^ ^7^ ^)^

Some studies evaluated the use of electronic prescriptions with the purpose of minimizing ME, and were carried out in several hospital settings. The majority, however, involved uncontrolled methods or used a control group not equivalent to the cases analyzed.^(^ ^8^ ^–^ ^10^ ^)^ Our hospital has four physically separated emergency departments (ED) that allow us to develop a controlled clinical study.

## OBJECTIVE

To analyze, in a controlled manner, the administration of medications to patients of four emergency departments in a single organization; in that, two units used the electronic medical record, and two, the conventional handwritten record.

## METHODS

A cross-sectional, retrospective, and observational study was conducted comparing ME in both scenarios of the same organization: the use of EMR *versus* the conventional handwritten record. All cases of ME reported in a one-year period were analyzed. The study was assessed by the organization Research Ethics Committee, opinion no. 452.994, CAAE: 20182113.7.0000.0071.

The ME were reported in all ED included in the study. The two ED that routinely used the conventional handwritten records were denominated Units 1 and 2, and the other emergency departments using the EMR, were called Units 3 and 4. These ED are physically independent, in different regions of the city of São Paulo (SP), but under management, administration, and coordination of similar teams with training and orientation of identical organizational protocols.

The similarity of the population seen at the four ED was marked by the major complaint upon admission, and the productivity of the units, that is, patients per physician, nursing team and pharmacist of the ED.

All cases reported by means of the electronic recording of events were included and duly investigated by the department in charge. The entire initial ME notifications that did not confirm having a ME as root cause were excluded from the investigation.

To evaluate the adverse events, we compared the number of ME per million opportunities (DPMO - defects per million opportunities). We compared these occurrences between the two groups. The process phase in which the failure happened and led to the ME and its severity was classified according to the NCC MERP^(^ ^1^ ^)^ and is presented on table 1.

**Table 1:** Classification of the National Coordinating Council for Medication Error Reporting and Prevention (NCC MERP)

	NCC MERP Classification
1	Wrong medication
2	Wrong dose
3	Error in preparation/handling
4	Prior history of allergies
5	Wrong concentration/dilution
6	Not administered
7	Wrong patient
8	Wrong infusion rate
9	Wrong route
10	Wrong frequency − delay in administration
11	Wrong administration technique
12	Unauthorized administration of the medication
13	Medication is contraindicated for the patient
14	Medication with expired validity date / deteriorated

Source: translated and adapted from National Coordinating Council for Medication Error Reporting and Prevention (NCC MERP). About medication error. What is a medication error [Internet]. 2001 [cited 2005 Jan 10]. Available from: https://www.nccmerp.org/about-medication-errors

The rate of ME was calculated based on the ratio between the number of reported ME by the total number of patients eligible in each organization, multiplied by 1 million, so that the measurement was in DPMO. The types of ME and diagnoses were described as absolute frequencies and percentages. The comparison between the groups as to error rates was done descriptively, by calculating the total number of ME during the period studied, and inferentially, by comparing the monthly rate of ME, in each group, using the Mann-Whitney's non-parametric test.

## RESULTS

The confirmation of the similarity between the different ED was marked by means of an analysis of the type of diseases treated in each one (Table 2).

**Table 2:** Profile of the units as to The International Statistical Classification of Diseases and Related Health Problems (ICD-10)^(^ ^11^ ^)^

Diagnosis	Unit 1	Unit 2	Unit 3	Unit 4
Not informed	8.27	3.33	6.53	6.02
J06 − Acute upper respiratory infections of multiple and unspecified sites	7.09	4.98	7.68	5.37
J01.0 − Acute maxillary sinusitis	4.20	0.03	4.94	3.90
J03.9 − Acute tonsillitis, unspecified	3.72	2.84	5.12	4.80
J02.0 − Streptococcal pharyngitis	3.51	0.03	2.56	1.66
J11.0 − Influenza with pneumonia virus not identified	2.97	1.57	1.33	0.35
A09.0 −Diarrhea and gastroenteritis of presumed infectious origin	2.96	5.00	5.30	4.81
M54.5 − Low back pain	2.20	1.90	1.42	2.02
N39.0 − Urinary tract infection, site not specified	1.89	2.14	2.06	1.79
R51.0 − Headache	1.87	1.19	1.68	2.34
R10.0 − Acute abdomen	1.47	0.02	1.22	3.16
R05.0 − Cough	1.21	0.93	2.22	4.05
H66.9 − Otitis media, unspecified	1.18	2.29	2.58	0.81
J06.9 − Acute upper respiratory infection, unspecified	0.61	4.60	1.43	2.15

Results expressed as percentage.

The units presented with the same complexity profile of the patients. The workload per healthcare professional (physician, nurses, and pharmacist) for each ED is presented on table 3.

**Table 3:** Workload per healthcare professional category

Patients per professional category	Unit 1	Unit 2	Unit 3	Unit 4	Total
Patients seen during the period	50,534	151,130	51,509	73,844	327,017
Patients per physician/month	122.16	144.44	141.12	148.03	140.72
Patients per nursing team/month	88.37	83.37	96.22	114.52	91.73
Patients per pharmacist/month	692.25	2,070.27	705.60	867.05	1,075.12

Unit 2 had the largest number of patients seen during the period and per pharmacist/month, and Unit 4 had the largest number of patients per physician and per nursing team. The distribution of ME events reported as per the NCC MERP classification during the period of 12 months is presented on table 4.

**Table 4:** Reported ME events according to the National Coordinating Council for Medication Error Reporting and Prevention (NCC MERP) over the period of 12 months

NCC MERP Classification	Unit 1	Unit 2	Unit 3	Unit 4	Total	%	p value
Wrong medication	0	11	0	1	12	27.27	0.037
Wrong dose	2	4	1	0	7	15.91	0.262
Error in preparation/handling	0	2	1	1	4	9.09	0.640
History of allergies	0	4	0	0	4	9.09	0.030
Wrong concentration/dilution	0	2	0	0	2	4.55	0.527
Not administered	0	2	0	0	2	4.55	0.527
Wrong patient	0	0	0	2	2	4.55	0.147
Wrong infusion rate	0	2	0	0	2	4.55	0.527
Wrong route	0	0	1	1	2	4.55	0.147
Wrong frequency − delay in administration	0	2	0	0	2	4.55	0.527
Wrong administration technique	0	0	1	1	2	4.55	0.147
Unauthorized administration of the medication	0	1	0	0	1	2.27	>0.999
Medication contraindicated for the patient	0	1	0	0	1	2.27	>0.999
Medication with expired validity date/deteriorated	0	0	1	0	1	2.27	>0.999
Total	2	31	5	6	44		0.069a
Partial total per type of patient record	33 (75.00)		11 (25.00)		44		
Patients seen per period	50,534	151,130	51,509	73,844	327,017		
Partial total DPMO per type of patient record	164 (65.07)		88 (34.93)		252 (100.00)		

Results expressed as n or n (%). DPMO: defects per million opportunities.

We found a larger number of ME in the units with the conventional records as compared to those with EMR (33 *versus* 11, respectively). The number of patients seen at units with conventional records was greater than with EMR.

In units that worked with the conventional records, the type of medication, dose, and administration to allergic individuals were the most frequent MEs, and in the units with EMR, the most common MEs were related to preparation/handling, wrong patient, wrong route, and wrong administration technique.

## DISCUSSION

Prevention of ME depends on processes implemented in the professional culture and their monitoring, helping to control these events. The most often used methodology for this is the “5 correct,” modified to “9 correct,” which is based on checking before administering: medication, patient, access route, dose, correct time - besides the four additional items: correct time, approach, validity, and documenting.^(^ ^4^ ^)^

As to productivity per healthcare professional category, the number of cases seen at an ED that used conventional records was greater than in the unit that had EMR; that is, there was also a greater possibility of a ME, which could have influenced in its greater occurrence when compared to EMR. Nevertheless, the number of patients per unit is also accompanied by greater capacity to receive them, with a larger nursing team for administration of medications, as was verified in the productivity of each ED. Taking into account that the teams of all emergency departments were selected and trained in the same way, and follow the same safety protocol, the ED with EMR had a noteworthy smaller incidence of events, which can suggest that the implementation of EMR in the other units would help in reducing ME.

The use of EMR is amply recognized as a component that enables access of the multidisciplinary team to the patient's data, which contributes to improvement of communication among these professionals, helps in clinical decisions, and improves compliance with the use of some primordial medications in certain clinical situations.^(^ ^10^ ^)^

It is also related to patient's safety, since it allows better quality of information and greater pharmacovigilance due to traceability enabled by the system.^(^ ^8^ ^)^ In systematic reviews, publications demonstrating effective improvement of the time spent by nurses for recording procedures in the medical records were identified. When the equipment is available at the bedside, time spent is reduced by 24.5%, but when it is kept in a central location, it was by 23.5%.^(^ ^9^ ^)^

According to what is described in the literature, units with EMR (3 and 4) demonstrated lower ME rates as compared to the units that use conventional records (1 and 2). The difference was not statistically significant (p=0.773) and that could be justified by the low number of ME, which lowers the statistical relevance of this datum.

One of the tools that the EMR system provides is safety warnings at different steps of the prescription, separation, and administration of the medication, similar to the results obtained at Australians organizations with the implementation of EMR.^(^ ^8^ ^,^ ^10^ ^)^

It is possible to perceive that when ME occur in the units with EMR, they are mostly related to preparation/handling of medication, wrong patient, wrong route, and wrong administration technique, as per the NCC MERP classification,^(^ ^1^ ^)^ which are errors related to the health professional's attention to the medication administration procedures.

On the other hand, in the units with conventional records, ME are more frequently associated to wrong medication, wrong dose, history of allergies, types of ME related to the legibility of prescription and its incomplete filling out, in which the most critical point is the absence of information regarding allergies to medications. This type of error can be blocked by the electronic digital system managed by pharmacists. These professionals can block or alert that what was prescribed with a wrong administration route or wrong dose, besides notifying the prescriber of the error made,^(^ ^12^ ^,^ ^13^ ^)^ requiring the correction of the prescription before its dispensing. Additionally, the verification of allergies and the patient's weight is mandatory, for greater safety of the process of medication administration.

In this study, the adverse errors related to the use of medications were present more frequently in the age range from zero to 9 years, similar to what was found in an American study.^(^ ^8^ ^)^ There were no cases of elderly patients, despite the frequent use of several medications, and consequently, an increased chance of error.^(^ ^14^ ^,^ ^15^ ^)^ More robust analyses with larger numbers of patients and events need to be conducted to show that the EMR contributes towards ongoing improvement in patient safety.

## CONCLUSION

The use of electronic medical record at emergency departments units was associated with lower rates of medication errors in this study.
